# Influence of the administration route and dose on the expression and antibody responses of a reporter and avian influenza self-amplifying mRNA vaccine in poultry

**DOI:** 10.1080/01652176.2025.2603307

**Published:** 2026-01-06

**Authors:** Janne Snoeck, Xiaole Cui, Pieter Vervaeke, Niek N. Sanders, An Garmyn

**Affiliations:** a Department of Pathobiology, Pharmacology and Zoological Medicine, Faculty of Veterinary Medicine, Ghent University, Merelbeke, Belgium; b Laboratory of Gene Therapy, Department of Veterinary and Biosciences, Faculty of Veterinary Medicine, Ghent University, Merelbeke, Belgium

**Keywords:** Self-amplifying mRNA (saRNA), mRNA vaccination, broiler chicken, administration route, dose-response effect, single-shot vaccination, prime-boost schedule, avian influenza, H5N1

## Abstract

Vaccination is routinely used in industrial poultry to control infectious diseases. Vaccines based on mRNA and self-amplifying RNA (saRNA) are approved for human use, but research on their application in poultry is limited. In this study the saRNA vaccine platform is evaluated in poultry. First, a luciferase-encoding saRNA (luc-saRNA) was tested as a model vaccine across different administration routes and doses in broilers. High luciferase expression, and anti-luciferase antibodies were observed after intramuscular (IM), subcutaneous (SC), and in ovo (IO) administration. After a second Luc-saRNA injection, seroconversion rates and antibody titers increased in the IM and SC group to almost 100%. Higher doses of Luc-saRNA increased luciferase production. However, they did not linearly increase antibody production, as all tested doses (0.20–5.0 µg) elicited an equipotent immune response. A vaccination experiment with saRNA encoding the hemagglutinin head-domain (HA-HD) of H5N1 avian influenza showed hemagglutinin inhibition (HI) titers that are indicative for protection after a single injection and these titers remained above the protective threshold during 6 weeks without boosting. When boosted, the HI titers increased four-fold. This study confirms effective protein translation and immune response induction in chickens with IM or SC administered saRNA-LNPs, even at the lowest dose of 0.20 µg.

## Introduction

Infectious diseases are a major concern in our modern poultry industry, impacting poultry health and welfare, economic outcomes such as pricing of poultry products and farm profitability, but also public health in case of zoonotic pathogens (Stewart-Brown n.d.; Meeusen et al. 2007; Abdul-Cader et al. 2018). While the effect on poultry health is evident through health decline and possible morbidity and mortality, the financial effect is caused by production losses including poor weight gain or productivity, condemnation of product, medications costs and inability to trade nationally and internationally (Meeusen et al. 2007; Abdul-Cader et al. 2018). Furthermore, a number of infectious avian pathogens are of additional global concern due to their zoonotic potential, their potential to evolve into more virulent variants (e.g. influenza viruses and Salmonella) and their ability to cross geographical boundaries and species (Brito et al. 2015). To minimize these risks, poultry farms adhere to multiple biosecurity measures, both external (e.g. farm entrance design and disposal of manure or dead animals) and internal (e.g. disinfection and anteroom management) (Ornelas-Eusebio et al. 2020; Delpont et al. 2021). However, routine vaccination of commercial poultry against a broad range of infectious diseases remains necessary.

At this point, most commercially available poultry vaccines are inactivated, live attenuated and viral vectored vaccines (Abdul-Cader et al. 2018; Leigh et al. 2018; Romanutti et al. 2020). For all intents and purposes, live and live attenuated vaccines aim to deliver an intact pathogenic genome to the individual in order to elicit an antigen-specific antibody response and a T cell response (Wrammert et al. 2009; Singanayagam et al. 2018). These vaccines are usually highly effective; however, in the case of live (attenuated) vaccines, there remains a potential—albeit generally low—risk of reversion to virulence, depending on the stability of the attenuating mutations. For example, attenuated infectious bursal disease virus and avian metapneumovirus strains have shown the capacity to regain virulence under certain field conditions (Cecchinato et al. 2014; Pikuła et al. 2018).

Hence, viral vector-based systems were developed as a potentially safer alternative (Brito et al. 2015). These vaccine platforms use a modified, non-pathogenic virus as a vector to deliver the genetic material of heterologous antigens from pathogens into host cells *via* naturally occurring, intracellular internalization pathways employed by the vector (Devlin et al. 2016). The two most commonly used poultry vaccine vectors are Turkey Herpes Virus (HVT) and Fowlpox Virus (FWPV), leading to development of vaccines against e.g. Newcastle Disease Virus (NDV), Infectious Bursal Disease Virus (IBDV), Infectious Laryngotracheitis Virus (ILTV) and *Mycoplasma gallisepticum.* While these vectors are considered safe and have not been associated with reversion to virulence, theoretical concerns about recombination between vector and wild-type viruses have been raised. However, such events are considered rare and have not been widely documented under field conditions, especially for widely used vectors like HVT (Brito et al. 2015). Additionally, anti-vector immunity, has been to effectively limit or even eliminate the efficacy of the viral vector vaccine (Romanutti et al. 2020; Klinkardt et al. 2024; Nissilä et al. 2025).

**Figure 1.**
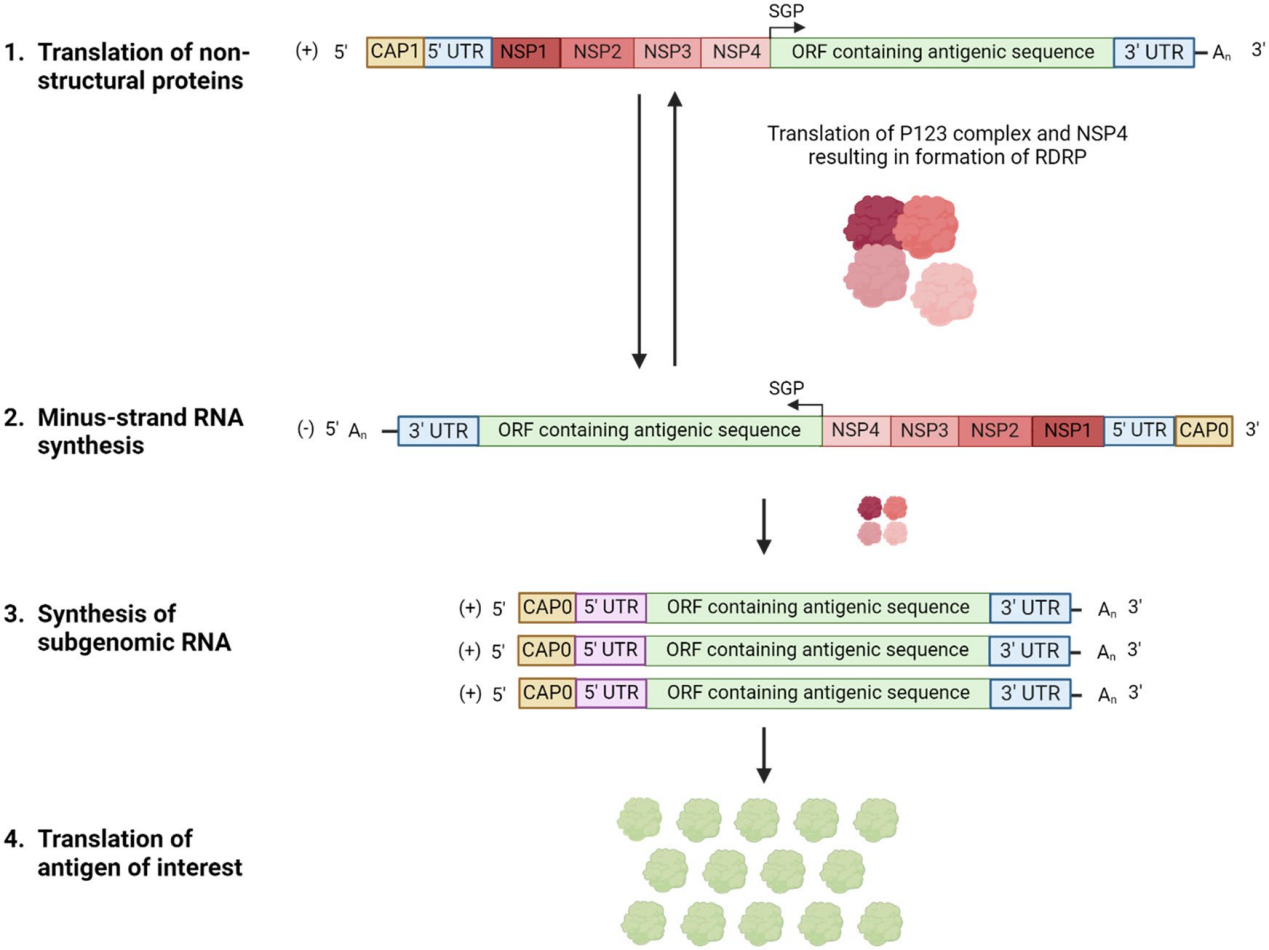
Schematic representation of the structure and the mechanism of the self-amplifying RNA (saRNA). The saRNA sequence consists of a capped 5′ untranslated region (5′ UTR), the open reading frame (ORF) encoding 4 VEEV-derived non-structural proteins (nsPs) encoding the replicase complex, a subgenomic promotor (SGP), the ORF encoding the antigen (in this paper either luciferase or H5) and the 3′ UTR followed by a poly(A) tail. The amplification mechanism of saRNA consists of 4 steps. (Stewart-Brown, 2015) Following delivery to the cytoplasm, the first ORF is translated, resulting in the production of the four NSPs. They are translated as the polyprotein P123 and separate nsP4, which form the early replicase complex. (Meeusen et al. 2007) The early replicase complex produces negative-sense saRNA. (Abdul-Cader et al. 2018) The replicase complex undergoes autoproteolytic cleavage to nsP1, nsP2, nsP3 and nsP4. The fully processed late replicate complex starts producing multiple positive-sense copies of both the entire saRNA as well as an excess of subgenomic RNA encoding the gene of interest, which in turn results in (Brito et al. 2015) in the translation of many copies of the antigen of interest.

Nonviral delivery of nucleic acid-based vaccines can evade said anti-vector immunity. DNA vaccination has been extensively researched in poultry, but did not lead to authorization for use in poultry due to limited immunogenicity and thus limited efficacy (Jazayeri and Poh 2019). Contrastingly, mRNA vaccines were among the most successful vaccines against SARS-CoV-2 during the COVID19 pandemic and have been administered to humans in a massive scale (Polack et al. 2020; Baden et al. 2021; Verbeke et al. 2021). mRNA vaccines consist of mRNA encoding the antigen(s) of interest and are usually encapsulated in a non-viral transfection vehicle (e.g. lipid nanoparticles (LNPs) or polymeric nanoparticles (PNPs)) (Guan and Rosenecker 2017). The mRNA can be either non-replicating mRNA or self-amplifying mRNA (saRNA), also known as replicon RNA. While non-replicating mRNA only encodes the antigen(s) of interest, self-amplifying mRNA also encodes a viral replicase which enables amplification of the RNA encoding the antigen (Figure 1). Most often, this replicase complex is alphavirus-derived (e.g. Venezuelan Equine Encephalitis Virus (VEEV), Sindbis virus or Semliki Forest virus) (Zhong et al. 2018). Hence, equivalent levels of protection can be achieved using lower saRNA doses compared to non-replicating mRNA, reducing the production cost of the vaccine (Vogel et al. 2018).

mRNA vaccination combines many positive attributes of the previously listed vaccines. mRNA vaccines elicit both humoral and cell-mediated immune responses and are unable to revert to virulence or risk genetic integration. Moreover, they are relatively simple to produce using robust, cell-free synthetic manufacturing methods that allow for rapid production (within days of obtaining gene sequence information), which makes them ideal for rapid responses to newly emerging pathogens or viral variants (Pardi et al. 2015, 2018; McKay et al. 2020; Baden et al. 2021; Baptista et al. 2021).

Despite these obvious advantages fundamental *in vivo* research into chicken mRNA vaccines or research into administration routes more suited for poultry has been investigated in detail so far. This study aims to investigate, elucidate and optimize the use of saRNA as a vaccine platform in chickens by administering an LNP-formulated saRNA reporter vaccine encoding firefly luciferase (FLuc2) (Leyman et al. 2018; Huysmans et al. 2019a, 2019b; Mc Cafferty et al. 2021) across different poultry-relevant administration routes and doses. Humoral immune response against the weak luciferase antigen were determined. Subsequently, an saRNA vaccine encoding the hemagglutinin head domain of H5N1 avian influenza virus was used to vaccinate broilers and confirm the trends seen during the luciferase-based research.

## Materials and methods

### Production of luciferase-encoding saRNA, hemagglutinin-encoding saRNA, and formulation in LNPs

The self-amplifying RNA (saRNA) (Figure 1) was produced from a linearized plasmid (pDNA) by *in vitro* transcription (IVT). The plasmids are derived from VEEV strain TC-83 and contain VEEV derived-5′ and 3′ UTRs and promotors. Additionally, substitutions in the 5′ UTR (r.3a > g) and in nsP2 (p.Q739L) are present in order to decrease cytopathogenic effects upon administration. The coding sequence of the structural proteins was replaced by either the reporter gene firefly luciferase or the head domain of the hemagglutinin (HA) antigen of influenza A H5N1 (A/Anhui/1/2005(H5N1); accession number HM172104). The construct was subsequently codon optimized for use in chickens. *E. coli* DH5α bacteria were transformed and subsequently cultivated in liquid broth (tryptone (10 g/l; VWR International, Leuven, Belgium), yeast extract (5 g/l; Invitrogen Merelbeke, Belgium), sodium chloride (10 g/l; VWR International, Leuven, Belgium)) containing ampicillin (0.1 mg/ml; PanReac Applichem ITW reagents, Darmstadt, Germany). Subsequently, the plasmids were isolated from these bacteria using the Plasmid Plus Midi kit (QIAGEN, Hilden, Germany) and was linearized using the I-SceI restriction enzyme (New England Biolabs, Ipswich, MA, USA). IVT was performed using the MEGAscript T7 Transcription kit (Life Technologies, Merelbeke, Belgium). SaRNA was co-transcriptionally capped with a cap1 structure (CleanCap AU (TriLink Biotechnologies, San Diego, CA, USA); 100 mM). Following IVT, the saRNA was treated with DNase I (Invitrogen, Merelbeke, Belgium) in order to remove template DNA and then purified using the Monarch RNA Cleanup kit (New England Biolabs, Ipswich, MA, USA). Additionally, cellulose purification was performed as described previously in order to remove double-stranded RNA (Zhong et al. 2021). The saRNA was stored at −80 °C. SaRNA integrity was evaluated using gel electrophoresis as described by Aranda et al. (Aranda et al. 2012). Once the quality of the saRNA was confirmed, the luciferase-encoding saRNA was formulated into LNPs by Acuitas Therapeutics (Vancouver, BC, Canada). Upon return, the functionality of said saRNALNPs- was evaluated by comparative transfection of HeLa cells against Lipofectamine Messenger Max (2:1) (Thermo Fischer Scientific, Invitrogen, Merelbeke, Belgium). The size, polydispersity index and zeta potential of the luc-saRNALNPs were determined- using a Nano ZS90 (Malvern Pananalytical, Worcestershire, UK) and were, respectively, quantified at 89 nm ± 16 nm, 0.137 ± 0.016 and −6.2 mV ± 0.5 mV (mean ± standard deviation). Calculations regarding these parameters employed a refractive index of 1.330 and a viscosity of 0.8872 mPa.s.

The HA-encoding saRNA was encapsulated in-house. LNP components included ALC-0315 (MedChemExpress; Monmouth Junction, NJ, USA) as the ionizable lipid, 1,2-dioleoyl-sn-glycero-3-phosphoethanolamine (DOPE) (Avanti Polar Lipids; Alabastar, AL, USA), 1,2-dimyristoyl-sn-glycero-3-methoxypolyethyleneglycol 2000 (DMG-PEG2000) (Avanti Polar Lipids; Alabastar, AL, USA) and cholesterol (Avanti Polar Lipids; Alabastar, AL, USA). Lipid components were dissolved in ethanol at the following molar ratio (ALC-0315: 50%; DMG-PEG2000 1.5%; cholesterol: 38.5%; DOPE: 10%) and saRNA was dissolved in NaOAc buffer (7.5 mM, pH 4.5; ThermoFischer; Merelbeke, Belgium). These two phases were thoroughly mixed together in order to allow sufficient RNA encapsulation. The mixture was dialyzed overnight at 4 °C in PBS (pH 7.4) using a Slide-A-Lyzer Dialysis Cassette 20 K MWCO (Thermo Fisher Scientific, Waltham, MA, USA). Encapsulation efficiency was determined by the Quant-it RiboGreen RNA Assay Kit (Thermo Fisher Scientific, Waltham, MA, USA) per manufacturer’s instruction. The size, polydispersity index, and zeta potential of the saRNA-LNPs were determined using a Nano ZS90 (Malvern Pananalytical, Worcestershire, UK). The size and zetapotential of the in-house saRNA-LNPS were, respectively, quantified at 146.5 nm ± 4.7 nm and −3.0 mV ± 1.1 mV (mean ± standard deviation). Ribogreen assay showed 80% encapsulation efficiency. Calculations regarding these parameters employed a refractive index of 1.330 and a viscosity of 0.8872 mPa.s.

### Experimental animals

One-day-old commercial ROSS 308 broilers chicks or eighteen-day embryonated ROSS 308 broiler eggs were purchased from a local hatchery (Vervaeke-Belavi, Tielt, Belgium). The embryonated eggs were hatched using a Covina Super incubator (Aveve, Wilsele, Belgium) at 37 °C. Chicks were housed on the floor covered with wood shavings in a climate-controlled facility with ad libitum access to water and feed (Versele Laga FARM mash 1 during the first ten days and mash 2 for the remainder of the experiment (Versele-Laga, Deinze, Belgium)). The experiments were conducted with the approval of the ethical committee of the Faculty of Veterinary Medicine, Ghent University (EC 2019/32 and EC 2020/03).

### Administration of LNP formulated luciferase-encoding saRNA to broiler chickens

*In vivo experiment 1: Evaluation of potential administration routes in terms of saRNA translation and humoral immune response*

One-day-old commercial ROSS 308 broiler chicks or eighteen-day embryonated ROSS 308 broiler eggs were randomly allocated to 6 groups of 6 animals each and received 1.0 µg of luciferase-encoding saRNA-LNPs (luc-saRNA-LNPs). The saRNA-LNPs were administered using four different administration routes, either intramuscular injection (IM) in the pectoral muscle, subcutaneous injection (SC) in the neck, ocular eye drops (OC) or *in ovo* injection (IO). Both dosage and formulation volume was fixed for all administration routes at 1.0 µg saRNA formulated in LNPs and diluted in 50 µl sterile, RNase free PBS (pH 7.4; contains 137 mM NaCl, 2.7 mM KCl, 8 mM Na_2_HPO_4_, and 2 mM KH_2_PO_4_; Invitrogen, Waltham, MA, USA). All chickens received a second administration on day 14 using their respective administration route (*n* = 6), except the IO group, which was boosted subcutaneously. Control groups were mock treated and received sterile PBS.

*In vivo* bioluminescence imaging was performed as described below at 6h, 1, 2, 3, 6, 9, 13 and 16 days post prime administration except in case of the *in ovo* group. In the IO group, first bioluminescence measurement was performed 6 h post-hatch, aligning the measurements with the other groups.

**Figure 2.**
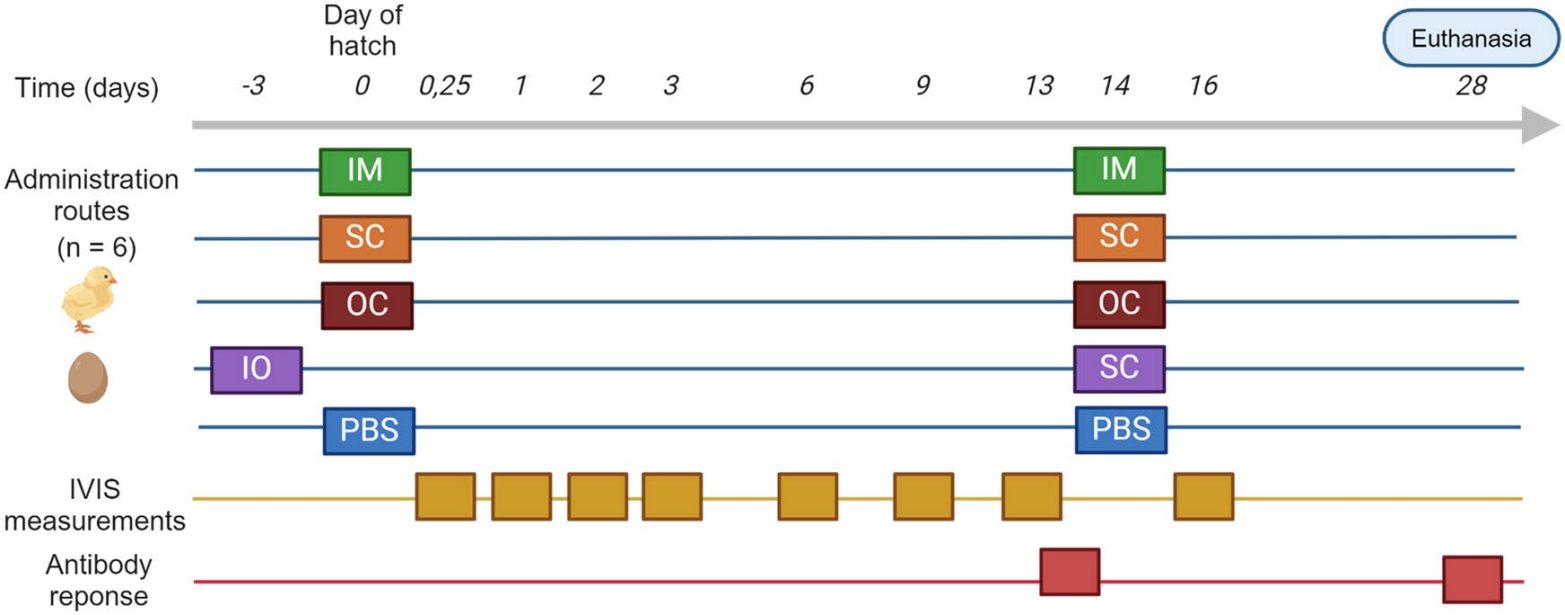
Schematic timeline of the *in vivo* trial evaluating saRNA expression and antibody titers after administration of 1.0 µg luciferase-encoding saRNA-LNPs in broiler chickens using different administration routes. Primo administrations with luciferase-encoding saRNA-LNPs were conducted either intramuscularly (IM), subcutaneously (SC), ocularly (OC) or in ovo (IO). saRNA-LNPs were administered again after 2 weeks using the same administration route, except for the IO group, which received the second shot SC. Control groups received sterile PBS *via* the IM, SC, OC or IO route. *In vivo* bioluminescence imaging was performed 6h, 1, 2, 3, 6, 9, 13, and 16 days post prime injection. Serum was collected immediately before the second administration (d14) and euthanasia (d28).

In order to assess the suitability of the saRNA as a vaccine platform in poultry, blood was collected from the wing vein to determine antibodies against the luciferase reporter two weeks after both the first and the second administration. Subsequently, serum was extracted from the whole blood samples by a 15 min centrifugation step (1000 g). On day 28, all chickens were euthanized by intravenous injection of sodium pentobarbital (Euthanimal 400 mg/ml; Alfa Med Ltd, Cork, Ireland). A scheme of the experiment with the time points of imaging, serum collection and euthanasia can be found in Figure 2.

*In vivo experiment 2: Evaluation of dose-response and effect of a second administration of saRNA-LNPs*

One-day-old ROSS 308 chicks were randomly allocated into 6 groups of 12 chickens each. Next, a dose of 0.20, 1.0 or 5.0 µg luc-saRNA-LNPs, diluted in RNase free PBS, was administered either SC or IM. At 14 days of age, six chickens from each group received a booster using the same saRNA dose and administration route as used in the prime. Mock treated chickens (*n* = 6), injected with sterile PBS at day 1 and day 14 of age, were included as controls. Bioluminescent imaging, serum collection and euthanasia were performed as described in *in vivo* experiment 1. A scheme of the experiment with the time points of imaging, serum collection and euthanasia can be found in Figure 6.

### Bioluminescence imaging

**Figure 3.**
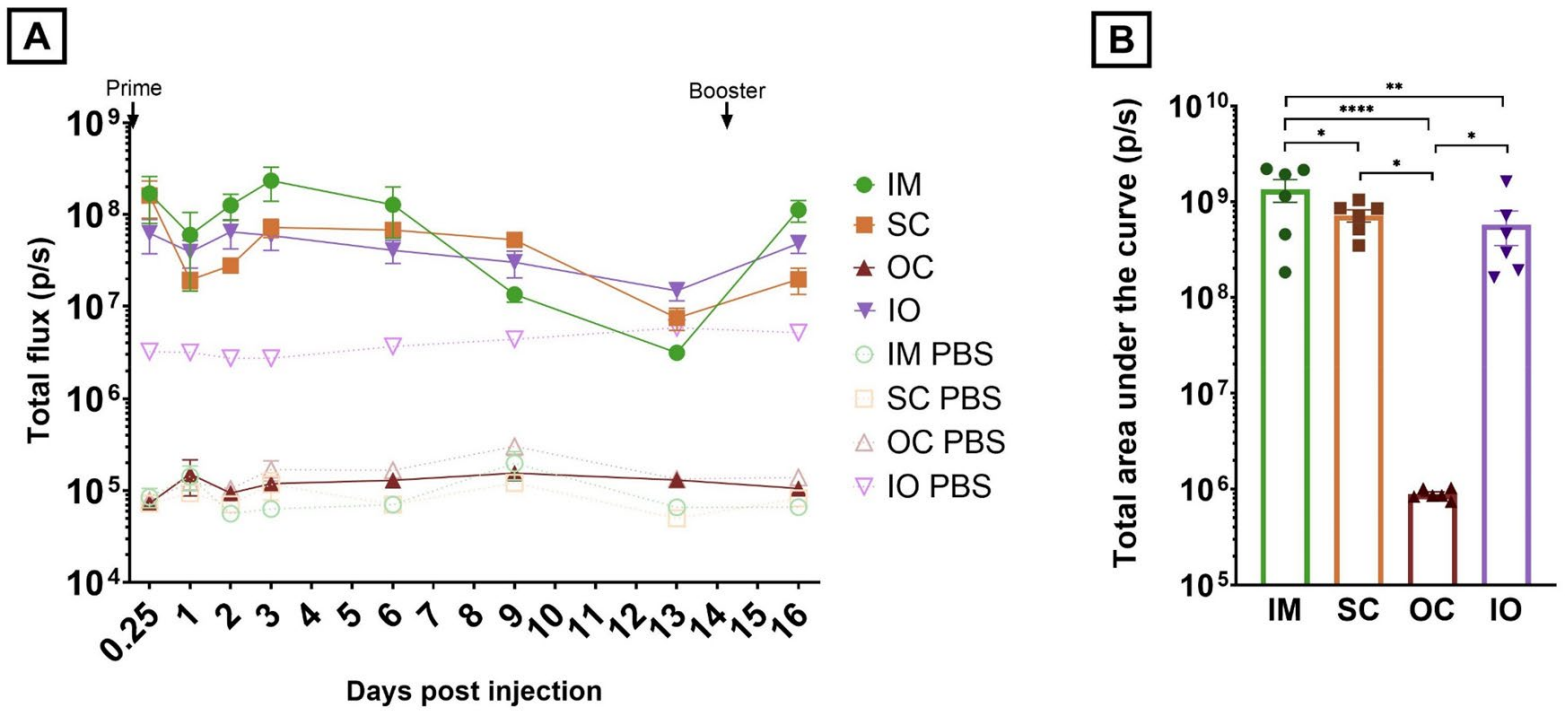
(A) Luciferase expression (total flux in photons per second (p/s)) over time following intramuscular (●), subcutaneous (■), in ovo (▼) and ocular (▲) administration of 1.0 µg saRNA-LNPs encoding firefly luciferase (*n* = 6). (B) The corresponding AUC normalized by subtraction of the respective background signal. IM and SC administration are significantly different from their respective negative controls at the 5% global significance level (Kruskal-Wallis) as estimated by measuring the area under the curve (AUC). Additionally, the difference between intramuscular, subcutaneous, in ovo and ocular administration, the difference between subcutaneous and ocular administration and between in ovo and ocular administration shows a clear difference in AUC (*p ≤ 0.05; **p ≤ 0.01; ***p ≤ 0.001; ****p ≤ 0.0001).

*In vivo* bioluminescence imaging of the chickens was performed using the IVIS Illumina III imaging system (PerkinElmer, Zaventem, Belgium). D-luciferin (PerkinElmer, Zaventem, Belgium) was administered SC at a dose of 150 mg/kg into the inguinal fold. Subsequently, anesthesia was induced at 5% isoflurane (Abbott Logistics, Breda, The Netherlands) and maintained on 2.5% isoflurane *via* a mask while being placed on the imaging platform. Kinetic studies revealed a bioluminescent plateau at 20 min post D-luciferin administration (data not shown). Hence, chicks were imaged 20 min post D-luciferin injection using an automated exposure time to ensure that the acquired signal was within effective detection range. Bioluminescence values were quantified by measuring photon flux in photons per second (p/s) in the region of interest where bioluminescence signal arose using the Living Image Software provided by PerkinElmer. The total amount of protein produced can be estimated by calculating the area under the curve (AUC). As luciferase catalytic activity has a half-life of 3 h *in vivo* (Thompson et al. 1991), its measurement reflects active translation of protein of the delivered saRNA. All reported AUC values are calculated including the last IVIS measurement at day 16, but without extrapolating the results until day 28. Signal background, determined by measuring the bioluminescent signal of control chickens treated with sterile PBS, was subtracted from the bioluminescent signal in order to be able to statistically compare to bioluminescent signal provided by each administration route. However, the graph displaying the expression data (Figure 3) shows the non-background subtracted data in order to correctly represent the difference between the research samples and the negative controls.

### IM Administration of LNP formulated HA-encoding saRNA to broiler chickens

One-day-old commercial ROSS 308 broiler were randomly allocated into 3 groups of 8 animals each. Two of these groups received 1.0 µg H5- encoding saRNA-LNPs in 50 µl RNase free PBS (pH 7.4; contains 137 mM NaCl, 2.7 mM KCl, 8 mM Na_2_HPO_4_, and 2 mM KH_2_PO_4_; Invitrogen, Waltham, MA, USA), whereas the third group received a mock formulation consisting of 50 µl of the same sterile, RNase free PBS. One of the vaccinated groups, referred to as the prime-boost group, received a second, booster administration on day 14. The other vaccinated group is referred to as the single-shot group. The control group received a second mock injection. Blood was collected from the wing vein to determine antibody production two, four and six weeks post hatch. Subsequently, serum was extracted from the whole blood samples by a 15 min centrifugation step (1000 g). On day 42, all chickens were euthanized by intravenous injection of sodium pentobarbital (Euthanimal 400 mg/ml; Alfa Med Ltd, Cork, Ireland). A scheme of the experiment can be found in Figure 10(A).

### Anti-luciferase antibody ELISA

Nunc Maxisorp^™^ high protein-binding plates (ThermoFischer Scientific, Waltham, MA, USA) were coated with 10 μg/mL recombinant firefly luciferase (Promega Corporation, Madison, WI, USA) for one hour at 37 °C. Plates were washed three times with washing buffer containing 0.05% Tween 20 (Sigma Aldrich, Saint Louis, MO, USA) in PBS (Gibco, Merelbeke, Belgium) and subsequently blocked overnight in blocking buffer containing 5% skim milk powder in PBS (Merck Millipore, Burlington, MA, USA) at 4 °C. Next, 100 µl of the serially diluted samples (1/10, 1/40, 1/160, 1/640) (blocking buffer) was incubated for 1h30 at RT. Afterwards, plates were washed 5 times and goat anti-chicken IgY Fc antibodies conjugated to horseradish peroxidase (ab112821; Abcam, Cambridge, UK) diluted 1:10 000 in blocking buffer, were added. After one hour at RT, plates were washed with PBS and 3,3′,5,5′-tetramethylbenzidine (TMB) (Biolegend UK Ltd., London, UK) substrate was added and incubated for 15 min. Afterwards, the reaction was stopped with stop solution (Biolegend UK Ltd., London, UK) and absorbance was measured in a microplate reader Biochrom EZ Read 400 Microplate Reader (Cambridge, United Kingdom) at 450 and 660 nm (reference wavelength). ELISA experiments were conducted in duplicate. ELISA cut-off values were defined as the average absorbance + three times the standard deviation of the absorbances of the corresponding negative control group.

### Hemagglutinin inhibition assay

The hemagglutinin inhibition (HI) assay was performed as described by Kaufmann et al. (Kaufmann et al. 2017). First, HA titration of the inactivated A/Anhui/1/2005 (H5N1) virus (NIBSC, Herts, UK) was determined using a 0.75% turkey blood cell suspension. Next, the serum was ¼ diluted with cholera filtrate incubated overnight at 37 °C, followed by 30 min incubation at 56 °C to inactivate cholera filtrate. 25 µl PBS was administered to each well of a 96 V-bottom plate. Subsequently, the first wells of each row received 25 μL of serum mixture and 2-fold serial dilutions were performed. The lowest dilution factor of serum is 8. Next, 25 μL of the inactivated strain matched H5N1 virus containing 4 HA units was added to each well. The plate was gently tapped and incubated for 30 min at room temperature. Finally, 50 μL of 0.75% turkey blood cell suspension was added to each well and incubate for 30 min. The plate was then tilted vertically for 25 s before read out.

### Statistics

Statistical analyses were performed using GraphPad Prism software (version 8.4.3, GraphPad, San Diego, CA, USA). Luciferase expression was analyzed post background subtraction using the respective negative control group. Quantitative luciferase expression was determined by using the area under the curve (AUC). A Kruskal Wallis test, the non-parametric variant of one way analysis of variance (ANOVA), was conducted. Correction for multiple comparisons (Dunn’s multiple comparisons test) was included. When comparing administration routes, each route was compared to one other. When comparing doses, each dose administered using IM injection was compared to each other.

Significant differences between ELISA and HI titers were determined by using a Kruskal Wallis test (nonparametric) as the conditions of normal distribution and equality of variance could not be guaranteed for each group. Correction for multiple comparisons (Dunn’s multiple comparisons test) was included. When comparing administration routes, each route was compared to each other. For statistical analyses of the effect of dosage and multiple saRNA administration, the antibody titers obtained after the first administration were compared to each dose and the negative control for each administration route (IM or SC) separately. The antibody titers obtained after the second administration (booster), albeit mock or saRNA, were compared to each dose and the negative control. Additionally, each mock-boosted was compared to their respective boosted group. No inter-administration route testing occurred in the dose-response trial. All statistical tests were performed at a global 5% significant level. The data are represented as means ± standard error of the mean (sem).

## Results

### Intramuscular, subcutaneous and in ovo administration of luciferase encoding saRNA-LNPs results in clear luciferase expression and anti-luciferase antibody production

Eighteen-day embryonated broiler eggs or one-day-old chicks (*n* = 6) were immunized with 1.0 µg luc-saRNA-LNPs using either the *in ovo* (IO), intramuscular (IM), subcutaneous (SC), or ocular (OC) route. All chickens, including the hatched chickens in the IO group, received a second injection on day 14 using the same delivery route as the first injection except for the IO group, which received the second injection SC (Figure 2). For each administration route, a control group was injected with PBS. As is commonly observed in broilers, two chickens in the control group died during the first week after hatch. One death could be attributed to heart failure, the other chick died due to umbilical infection (omphalitis). Mortality or disease symptoms were not encountered in any of the chickens receiving saRNA-LNPs.

Shortly after the first administration, the IM, SC and IO routes resulted in high expression that slightly dropped from day 3 onwards. In contrast, ocular delivery of saRNA-LNPs by eye drops did not induce significant expression (Figure 3).

The IM, SC and IO groups displayed similar expression kinetics. In detail, both the IO and SC routes resulted in a first expression peak 6 h (0.25 day) post hatch/prime injection, followed by a small decline and a subsequent new peak at 2 days post hatch (IO) or 3 days after the first injection (SC). After this second peak, the expression signal decreased. IM administration resulted in the highest expression peaks at 6h and 3 days post prime. However, a steeper decline of the expressed signal compared to the SC and IO group could be observed after the second peak. Thirteen days after the first administration, the bioluminescent signal in all three groups still largely exceeded the bioluminescent signal of the negative control group. After the second administration, the expression reached similar peak levels as after the first administration in all administration routes. Expression levels beyond 16 days of age could not be monitored because the chickens grew too large to fit in the imaging system.

**Figure 4.**
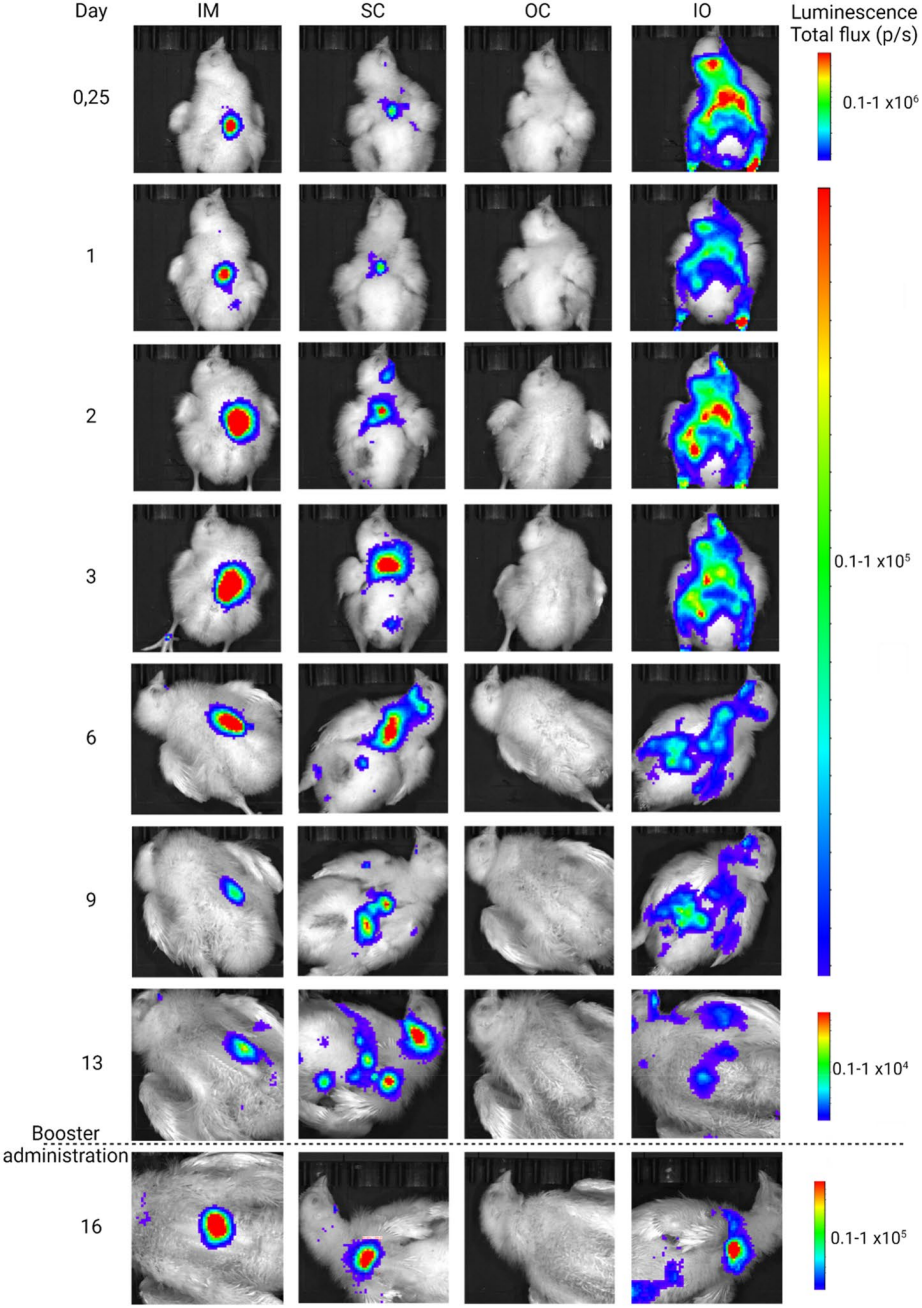
Bioluminescent images showing the biodistribution and duration of the luciferase expression after intramuscular (IM), subcutaneous (SC), ocular (OC) an *in ovo* (IO) administration of 1.0 µg of luciferase-encoding saRNA-LNPs in broiler chickens. The images at day 16 were obtained 2 days after administration of the boost. The images are representative IVIS images of a single broiler chicken. The luminescence signal (photons/second) is shown and the scale per pixel of said plot is indicated next to the images.

In contrast to the kinetics, the biodistribution of the expressed protein differs between the delivery routes. Bioluminescence imaging shows that SC and IM injection of saRNA-LNPs generates a local expression pattern near the injection site, whereas IO injection results in a more widespread, diffuse luciferase expression spread over the entire body (Figure 4). At day 16 however, the IO group displayed a localized expression pattern, which can be attributed to the SC administration of the booster. Still, leftover diffuse expression remains visible at day 16. The IO administered PBS-buffered control resulted in a higher background signal than the other routes (Figure 3A). This can be attributed to the presence of white feathers, which were not removed in the IO group due to the broad expression area. To adjust for this difference, the area under the curve (AUC), which is a measure of the cumulative protein production during the whole experiment, was adjusted to the specific background of each group.

At 1.0 µg, the AUC of the IM group resulted in the highest amount of total protein produced (1.3 × 10^9^), followed by the SC (7.2 × 10^8^), IO (5.7 × 10^8^) and OC (8.2 × 10^5^) group, respectively (Figure 3B). Luciferase expression after IM, SC and IO administration was significantly different from their respective negative controls (*p* ≤ 0.0001 and *p* = 0.0193 and *p* = 0.0170). Additionally, the AUC was significantly different between IM and OC administration (*p* ≤ 0.0001), SC and OC administration (*p* = 0.0195), IO and OC administration (*p* = 0.0389) and IM and IO administration (*p* = 0.0325).

**Figure 5.**
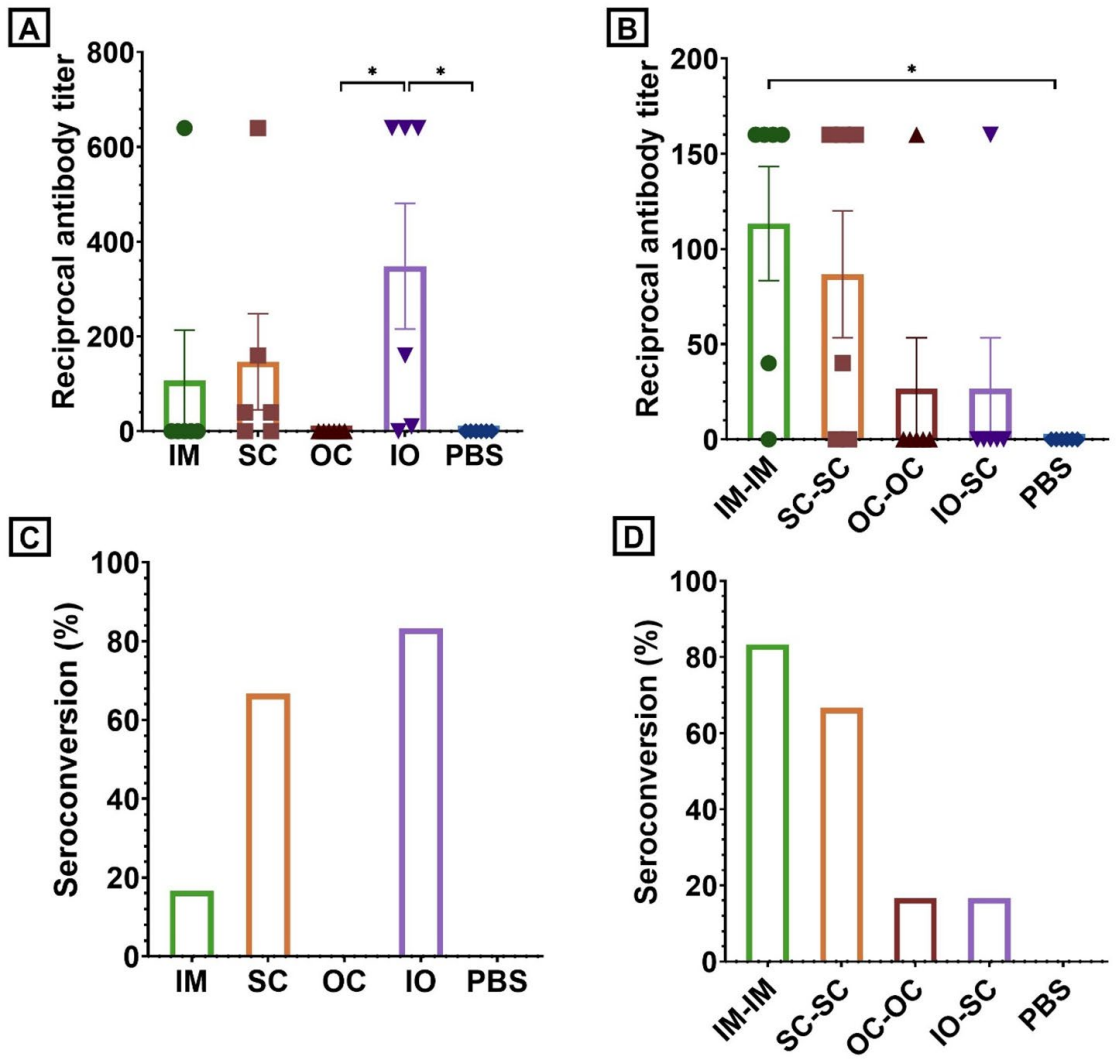
Luciferase-specific IgY antibody titers and corresponding seroconversion rates two weeks after prime vaccination (A–C) and two weeks after booster vaccination (B–D) following intramuscular (IM), subcutaneous (SC), ocular (OC) and *in ovo* (IO) administration of luciferase-encoding saRNA-LNP (*n* = 6). In order to allow comparison, serum samples of animals who received injections containing only PBS were included in the experiment. Following a Kruskal-Wallis test, the difference in anti-luciferase IgY titers turned out to be significant between the IO group and both the OC and the negative control group after prime vaccination. Following booster administration, the difference between the IM group and the negative control group was statistically significant (*p ≤ 0.05; **p ≤ 0.01; ***p ≤ 0.001; ****p ≤ 0.0001).

While these results answered the main questions for this experiment, we determined anti-luciferase antibodies as a secondary outcome. It should however be noted that administration of luciferase-encoding DNA is known to induce only low titers of anti-luciferase antibodies in mice, indicating that luciferase has only limited antigenic properties (Vandermeulen et al. 2007; 2009; Petkov et al. 2013). Keeping this in mind, serum samples of all chickens were collected two and four weeks post hatch. The first samples allow to estimate the efficacy of a single shot vaccination, whereas the serum samples collected on day 28 demonstrate the effect of a booster vaccination. Despite the low immunogenicity of luciferase, seroconversion could be observed in some animals after prime vaccination (IM: 17%, SC: 67%, OC: 0%, IO: 83%) (Figure 5). The corresponding titers were, respectively, 107, 147, 0 and 348. The difference between the IO group and both the OC and the PBS group turned out to be statistically significant (both *p*-values = 0.0253).

**Figure 6.**
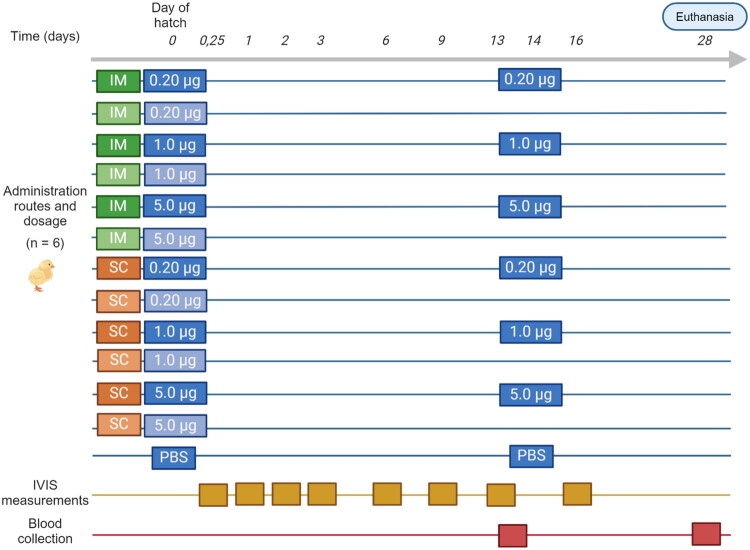
Schematic timeline of second *in vivo* broiler trial evaluating dosage and the need for a booster vaccine. Primo administrations were conducted either intramuscularly (IM) or subcutaneously (SC), employing a dose of either 5.0, 1.0 or 0.20 µg of saRNA. When boosters were given, second administrations were administered using the same administration route as the primo injection. Control groups received sterile PBS. *In vivo* bioluminescence imaging was performed 6h, 1, 2, 3, 6, 9, 13, and 16 days post prime injection. Serum was collected immediately before booster vaccine administration (d14) and euthanasia (d28).

After the second administration serum titers were unexpectedly lower than after the prime (IM: 113; SC: 87; OC: 27; IO: 27), while seroconversion rates increased in the IM group, remained the same in the SC group and decreased in the IO group (IM: 83%; SC: 67%; OC: 17%; IO:17%). Only the antibody titers in the IM group turned out to be significantly higher (*p* = 0.0398) than the titers in the negative control group, where no anti-luciferase antibodies were detected.

### Intramuscular administration of the lowest dose of luciferase-encoding saRNA-LNP results in the lowest luciferase expression yet induced the highest antibody titers

Next, the effect of saRNA dosage was evaluated. Based on the previous experiment, the IM and SC administration route were selected for the dose-titration experiment using 0.20, 1.0, or 5.0 µg of luc-saRNA-LNPs. On day 0, the chickens (12 per group) received their first saRNA-LNP administration. On day 14, serum samples were collected and half of the chickens (*n* = 6) received a second injection. Serum samples of all chickens were collected again on day 28. Bioluminescence was measured 6h, 1, 2, 3, 6, 9, 13, and 16 days post prime injection. One chicken (probably a non-starter) died two days post saRNA-LNP injection after receiving a single 0.20 µg dose. Besides cachexia and anorexia, significant lesions were not observed after postmortem evaluation.

**Figure 7.**
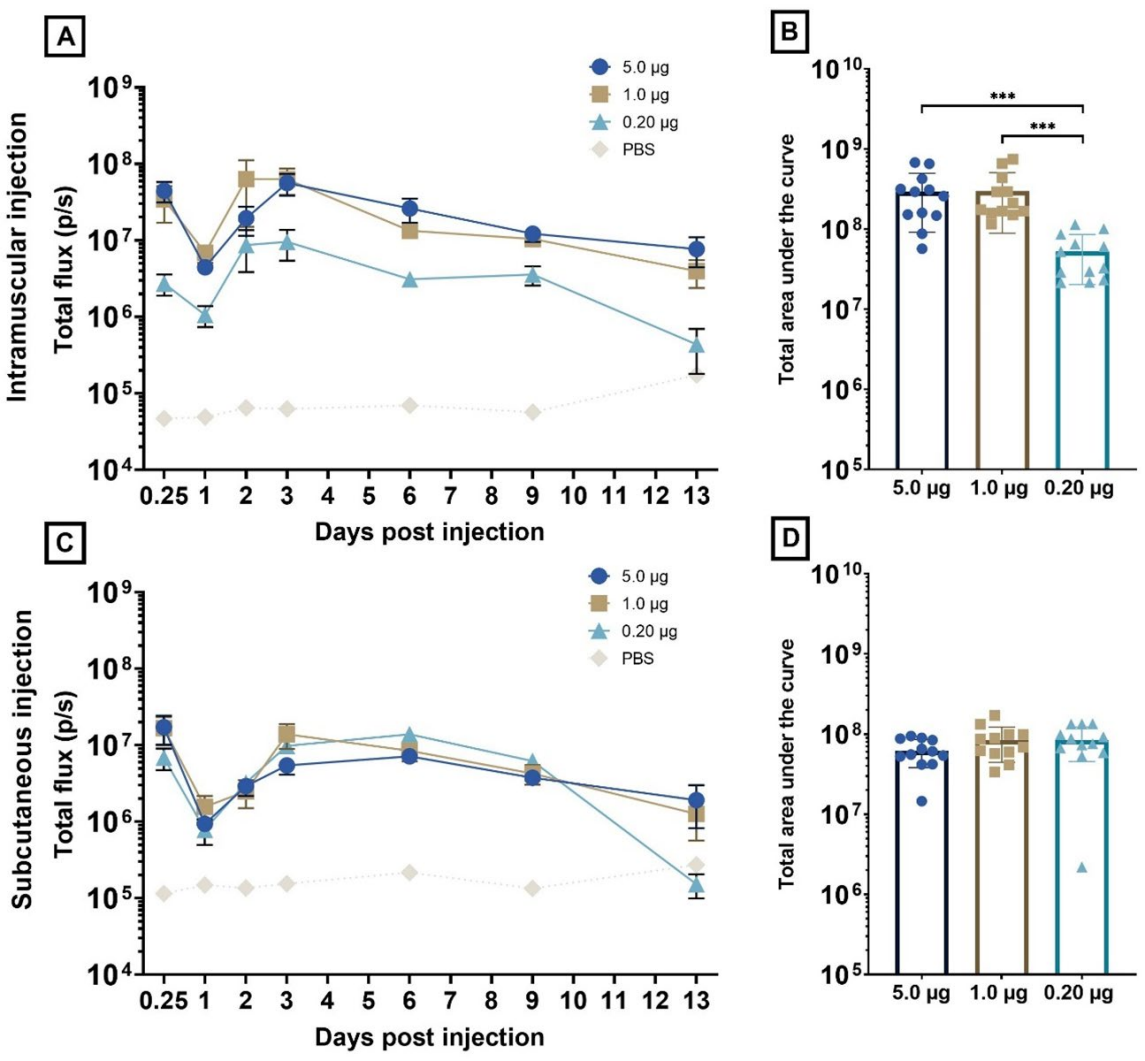
Luciferase expression (total flux in photons per second (p/s)) over time (from 6 h (0.25 days) to 13 days post administration) following intramuscular (A) and subcutaneous (C) injection of 5.0 µg (●), 1.0 µg (■), and 0.20 µg luciferase-encoding saRNA-LNPs (▲). (B–D) The corresponding AUC normalized by subtraction of the respective background signal. Following IM administration, the 5.0 and 1.0 µg dose significantly outperformed the 0.20 µg dose at the 5% global significance level (Kruskal-Wallis) when comparing the area under the curves. After subcutaneous administration, none of the doses differed significantly from each other (5.0 µg: *p* = 0.0118; 1.0 µg: *p* = 0.0050; 0.20 µg: *p* = 0.0234) (*p ≤ 0.05; **p ≤ 0.01; ***p ≤ 0.001; ****p ≤ 0.0001).

Each of the administered doses resulted in a clear luciferase expression with a kinetic profile similar to that observed during the first *in vivo* trial (Figure 7A). A dose of 0.20 µg saRNA-LNPs resulted in clear protein production with an AUC of 5.30 × 10^7^ (Figure 7B). Increasing the dose to of 1.0 and 5.0 µg resulted in statistically significant higher protein production (AUC, respectively, 2.98 × 10^8^ and 2.95 × 10^8,^ respective *p*-values: 0.0001 and 0.0004). Still, the additional increase is only 4-fold, which is somewhat surprising as the dose is increased 5-fold (1.0 µg;) or even 25-fold (5.0 µg).

Two weeks after the initial administration, the mean anti-luciferase antibody titers of the single-shot group were 110, 213, and 108 in, respectively, the 0.20, 1.0, and 5.0 µg dose group. The group meant to test the prime-boost setting resulted in titers of 213, 113, and 33 in, respectively, the 0.20, 1.0, and 5.0 µg dose group. No statistical difference between doses could be discerned after prime injection. Respective seroconversion rates were 50%, 33%, and 33% in the single-shot setting and 33%, 33%, and 33% in prime-boost setting (prior to boosting).

**Figure 8.**
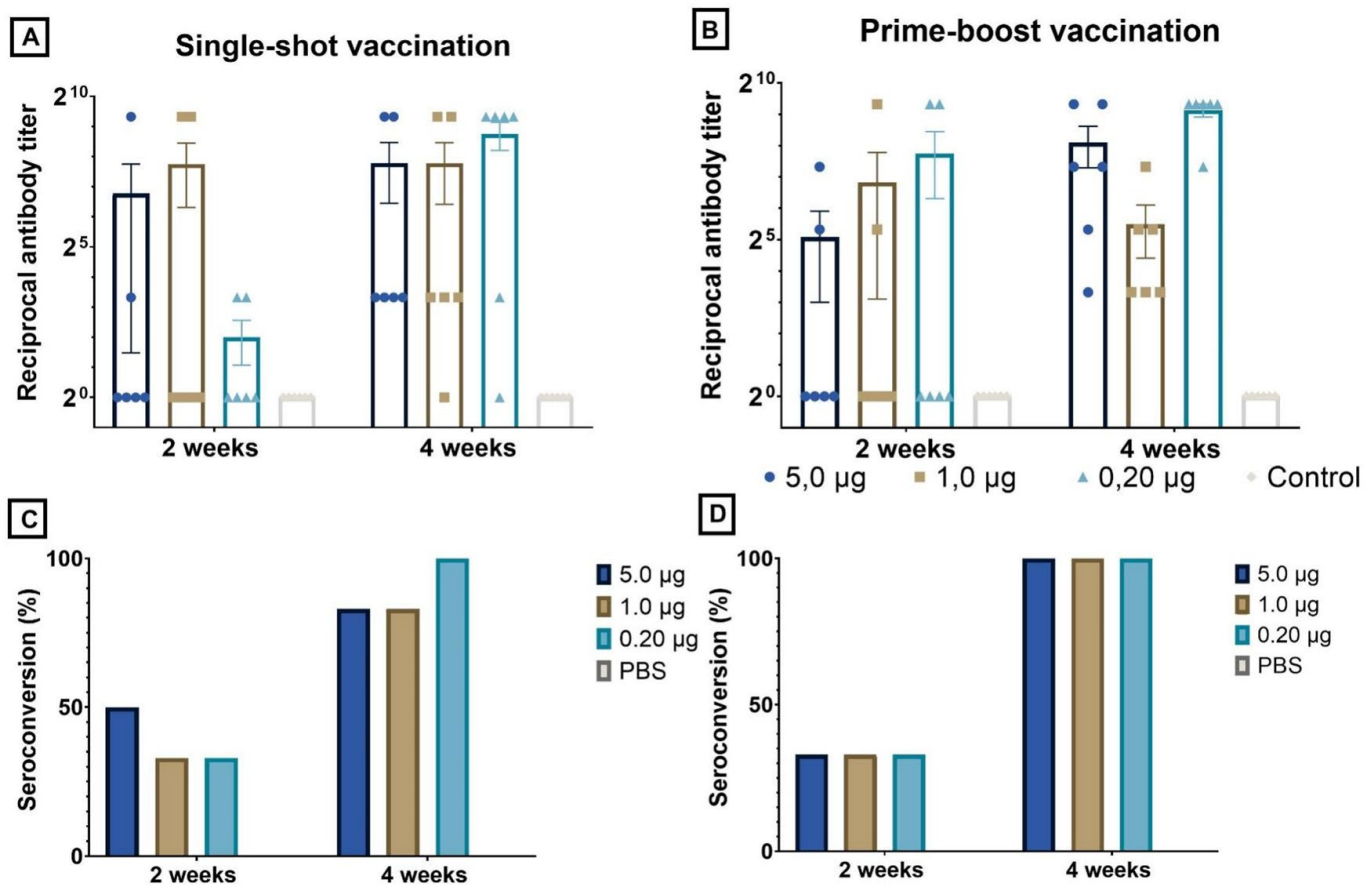
Anti-luciferase antibody (IgY) titers and corresponding seroconversion rates after IM single-shot administration (A) and IM prime-boost administration (B) of luc-saRNA-LNPs. Corresponding seroconversion rates can be found in panel (C) and (D). A dose of either 5.0 µg (●), 1.0 µg (■), or 0.20 µg (▲) of luc-saRNA-LNPs was administered. Additionally, a negative control group which only received sterile RNase-free PBS was included. Two weeks after both single-shot and prime-boost administration, no significant difference in antibody titers were observed between the different doses. However, four weeks after the initial administration (i.e. two weeks after the booster administration in the prime-boost group), three groups showed statistically significant results compared to the negative control: the prime-boost 0.20 µg group, the 0.20 µg single-shot group and the prime-boost 5.0 µg group. No statistical difference between vaccinated groups themselves could be discerned (Kruskal-Wallis; *p ≤ 0.05; **p ≤ 0.01; ***p ≤ 0.001; ****p ≤ 0.0001).

Subsequently, half of the chickens (*n* = 6) received a second, booster injection. Results of the anti-luciferase IgY antibody ELISA and seroconversion rates are visualized in Figure 8. Two weeks after the second administration, the mean anti-luciferase antibody titers were 560, 45, and 275 in, respectively, the 0.20, 1.0, and 5.0 µg boosted groups and 428, 218, and 220 in the single-shot groups which indicates that antibody titers in the single-shot vaccinated groups do not drop after two weeks but continue to increase. All boosted groups and the 5.0 µg single-shot group achieved 100% seroconversion, whereas the single-shot 0.20 and 1.0 µg groups showed an increase in seroconversion to 83% at day 28. Boosted groups generally showed both higher antibody titers and higher seroconversion rates compared to mock-treated groups, but it is noteworthy that the boosted 0.20 µg group resulted in the highest mean antibody titers. Statistical significant differences between the negative control group and 3 treated groups could be observed: the boosted 0.20 µg group (*p*-value = 0.0003), the single-shot vaccinated 0.20 µg group (*p*-value = 0.0003) and the boosted 5.0 µg group (*p*-value = 0.0209).

### Subcutaneous administration of luciferase-encoding saRNA-LNPs results in a dose-independent expression and higher antibody titers in the lowest dose groups

When administering the saRNA-LNP formulation subcutaneously, a dose of 0.20 µg resulted in the highest amount of total protein produced (8.4 × 10^7^), followed closely by a dosage of 1.0 µg (8.3 × 10^7^) (Figure 7C,D). A dose of 5.0 µg resulted in the lowest amount of total protein produced (6.2 × 10^7^). None of the doses differed significantly from each other, yet all of them differed significantly from the negative control (5.0 µg: *p* = 0.0429; 1.0 µg: *p* = 0.0015; 0.20 µg: *p* = 0.0005).

**Figure 9.**
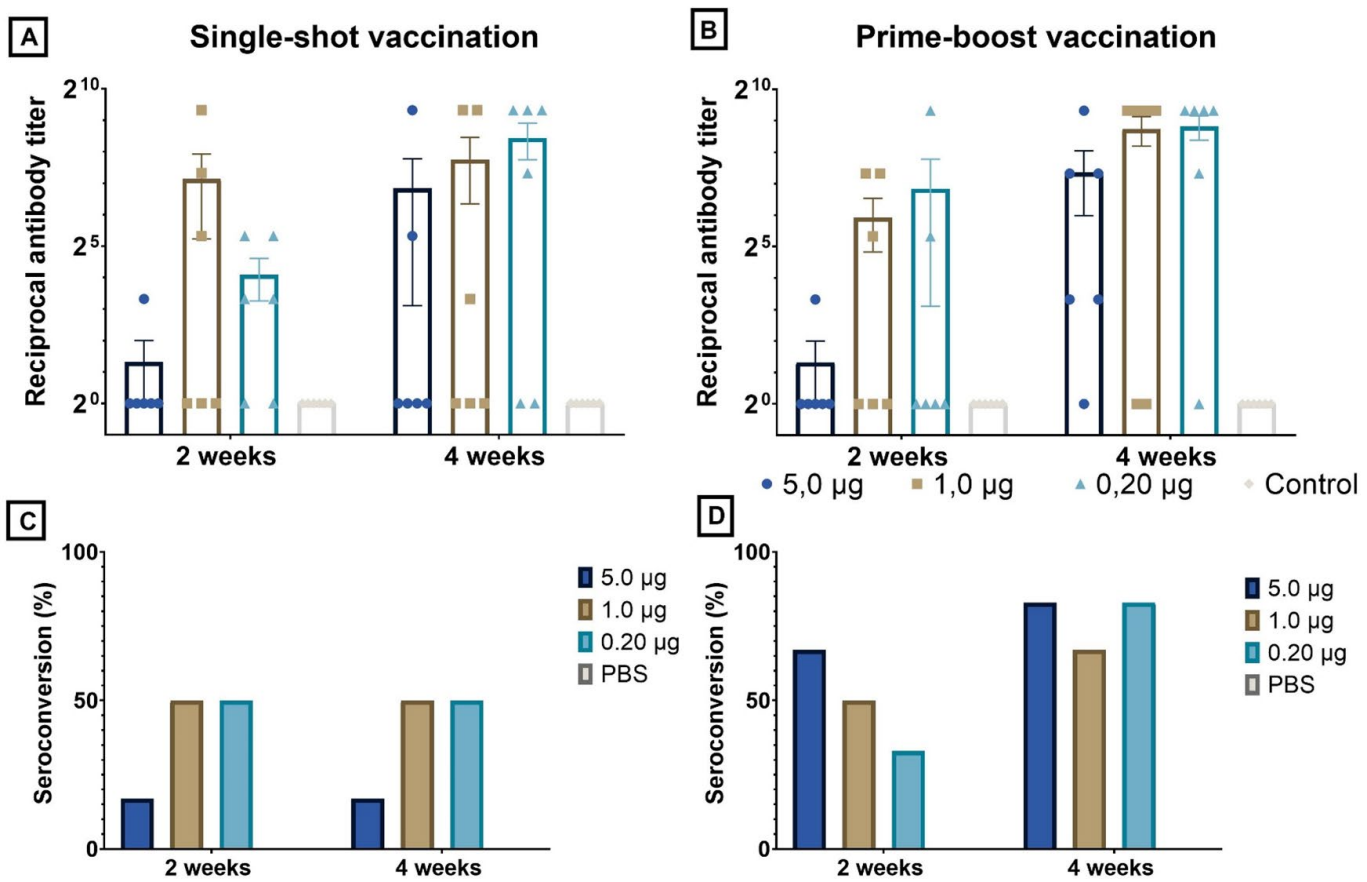
Anti-luciferase antibody (IgY) titers and corresponding seroconversion rates after SC single-shot administration (A) and SC prime-boost administration (B) of luc-saRNA-LNPs. Corresponding seroconversion rates can be found in panel (C) and (D). A dose of either 5.0 µg (●), 1.0 µg (■), or 0.20 µg (▲) of luc-saRNA-LNPs was administered. Additionally, a negative control group which only received sterile RNase-free PBS was included. Although the mean titers after prime injection were relatively low, a statistical difference between the 0.20 µg dose and the negative control and the 1.0 µg dose and the negative control could be found. Two weeks after the boost, all mean titers were higher than after a single prime vaccination, even those of the mock-boosted groups, yet no statistical difference between groups could be detected (Kruskal-Wallis; *p ≤ 0.05; **p ≤ 0.01; ***p ≤ 0.001; ****p ≤ 0.0001).

Two weeks after the first injection, the mean anti-luciferase antibody titers were 17, 140, and 2 in, respectively, the single-shot 0.20, 1.0, and 5.0 µg dosage group (Figure 9). The prime-boost group resulted in titers of 115, 60, and 2, respectively. The corresponding seroconversion rates in the single-shot group were 67%, 50%, and 50% while seroconversion rates in the prime-boost group were 50%, 50%, and 17%. Half of the chickens in each group were boosted at day 14. Four weeks after the single-shot vaccination, mean antibody titers were higher than after two weeks (0.20 µg: 348; 1.0 µg: 215; 5.0 µg: 113) and the seroconversion rates were 67%, 50%, and 33% in, respectively, the 0.20 µg, 1.0 µg and or 5.0 µg dosage group. Two weeks after the boost, mean titers were higher than the ones reported after a single-shot vaccination and equaled 453, 427, and 163, respectively, in the 0.20 µg, 1.0 µg and or 5.0 µg dosage group. Seroconversion rates also increased after the booster were 83%, 67%, and 83% in the 0.20, 1.0, and 5.0 µg group. After the boost, antibody titers were not significantly different between dosages, however, the 0.20 µg group showed borderline significant higher titers (*p* = 0.0510).

### Intramuscular administration of HA-encoding saRNA-LNPs results HI titers that are indicative for protection

**Figure 10.**
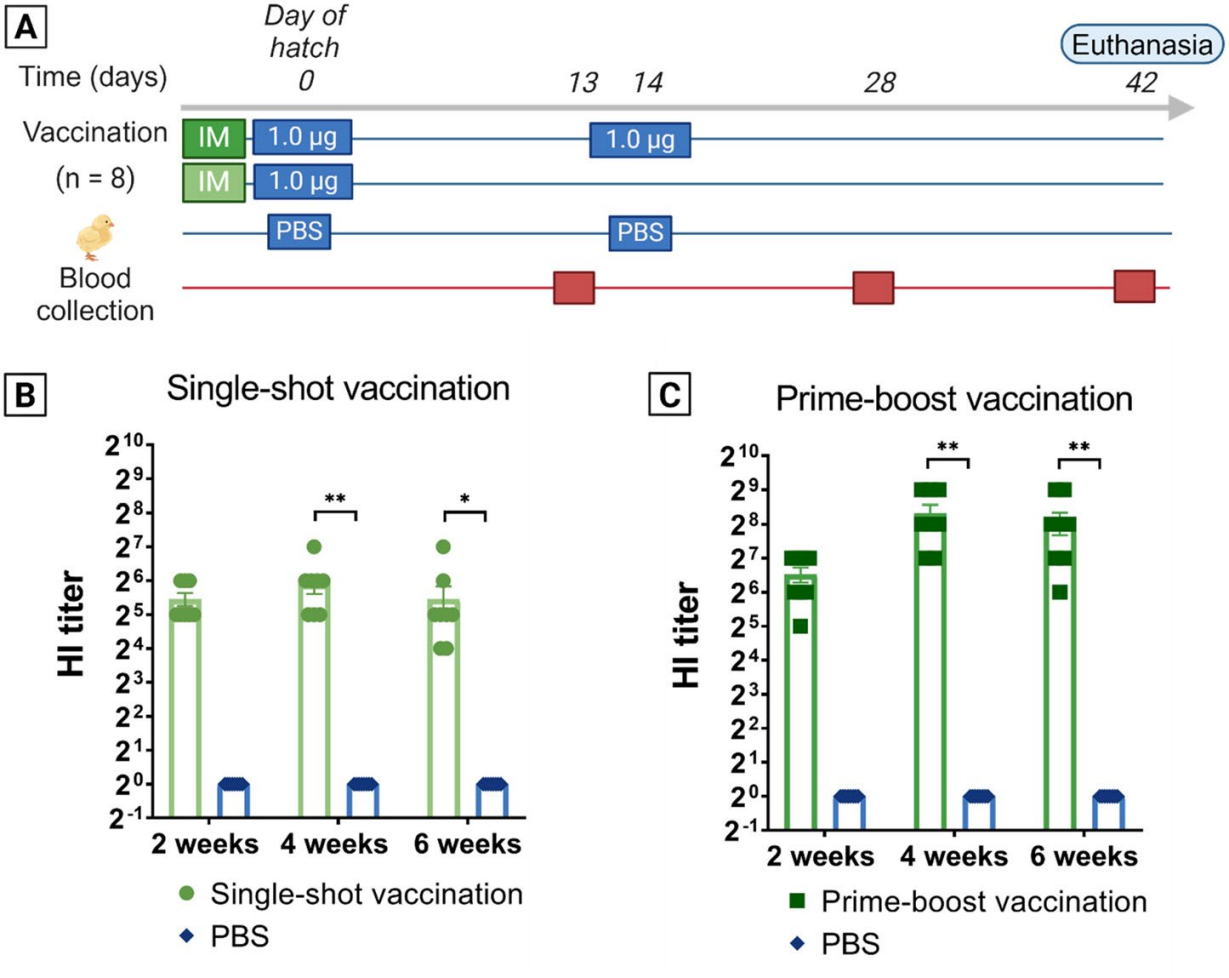
(A) Scheme and timeline of the *in vivo* trial evaluating antibody production and the need for a booster administration employing 1.0 µg saRNA-LNPs encoding the head domain of the H5 antigen of influenza A (H5N1). All administrations were conducted intramuscularly (*n* = 8). Prime administrations were conducted on the day of hatch, and a second administration occurred 2 weeks later. Control groups received sterile PBS. Serum was collected right before the second administration (d14), on day 28 and right before euthanasia (d42). (B, C) Resulting hemagglutinin inhibition (HI) titer in serum two, four and six weeks after (prime) vaccination with LNP-formulated saRNA encoding the H5 head domain. In order to allow comparison, serum samples of animals who received injections containing only PBS were included in the experiment as a negative control. Mean HI titers of the single-shot group were 44, 60 and 44 at, respectively, 2, 4, and 6 weeks, whereas the prime-boost group displayed titers of 92, 320 and 264. The statistical difference determined by Kruska-Wallis test, is indicated on the figure (*p ≤ 0.05; **p ≤ 0.01; ***p ≤ 0.001; ****p ≤ 0.0001).

To generate a proof-of-concept with an immunodominant antigen, saRNA encoding the secreted head domain of the HA antigen (HA-HD) of H5N1 avian influenza was produced. Chickens were IM immunized with 1.0 µg of H5-encoding saRNA encapsulated in in-house formulated LNPs. On the day of hatch, the chicks (*n* = 8) received the prime saRNA-LNP administration. Two weeks later (on day 14), serum samples were collected and one group, referred to as the boosted group, received a second injection. The second group, referred to as the single-shot group, received only the prime injection and was instead injected with a mock formulation on day 14. An additional negative control group received PBS at all moments of vaccination. Serum samples of all chickens were collected on day 28 and on day 42. No animals died during the course of this experiment. During statistical analysis, one outlier in the boosted group had to be removed from the data set as it deviated more than 3 times the standard deviation from the statistical mean. Experimental design is visualized in Figure 10A.

The mean HI titer of the single-shot shot group equaled 44, 60, and 44 at, respectively, 2, 4 and 6 weeks after vaccination, signaling both possible protection against mortality (titer > 32) and prevention of viral shedding (titer > 40) (Swayne and Sims 2021; Terrestrial Animal Health Code 2023; WOAH Terrestrial Manual 2025 2024) (Figure 10(B)). In the boosted group, the mean HI titer equaled 90, 320 and 264, respectively, 2, 4 and 6 weeks after vaccination (Figure 10(C)). The difference in mean HI titer between the negative control and single-shot vaccination group at 2 weeks was clear, yet is not statistically significant (*p* = 0.1206). Comparison between the negative control and the single-shot group at 4 weeks and 6 weeks led to the respective *p*-values of 0.0029 and 0.0317. When comparing the negative control and the boosted group at 4 weeks (*p* = 0.0026) and 6 weeks (*p* = 0.0077), the difference was statistically significant. The difference in HI titer between the single-shot group and the boosted group at 4 weeks and 6 weeks was significant (4 weeks: *p* = 0.0074; 6 weeks: *p* = 0.0183).

## Discussion

When designing a poultry vaccine, several crucial elements must be considered. The ideal vaccine is effective, limits infection, replication and transmission of the pathogen (Read et al. 2015). It should also be suitable for mass application in terms of administration route, vaccination schedule and pricing.

Poultry vaccines are often administered *via* drinking water, *in ovo* injection or whole body spray, with the latter mainly targeting the respiratory tract (Leigh et al. 2018). Subcutaneous administration is more labor intensive, but can be automated, lending itself to high-throughput vaccination as well (Cargil and Joey n.d.). Studies have shown that the administration route of live or live attenuated vaccines in poultry impact the immune response in chickens (Gough and Alexander 1979; Kembi et al. 1995; Bughio et al. 2017; Leigh et al. 2018) and the immunogenicity of mRNA vaccines in mice (Pardi et al. 2015). However, studies investigating the effect of delivery routes on the expression and efficacy of mRNA vaccines in poultry are lacking.

Recently, we established that LNP-delivered saRNA is successfully translated in chicken primary cecal cells, tracheal explants and conjunctival explants (Snoeck et al. 2023). This study confirmed effective delivery and entry into the cells and successful saRNA translation in chicken tissues and cells.

Current study looked further into the *in vivo* potential the saRNA platform in poultry.First, the IM, SC, IO and OC administration routes were evaluated at a dose of 1.0 µg. The IM, SC and IO administration resulted in clear *in vivo* luciferase expression, confirming translation of saRNA in multiple chicken tissues and cells. Ocular administration did not result in detectable luciferase surface expression. The latter was surprising as multiple studies using conventional vaccines reported the ocular administration route to be effective as several lymphatic tissues, such as the conjunctiva-associated lymphoid tissue and the Harderian gland, can be found near this immunization site (Beltrán et al. 2019; Al-Rasheed et al. 2021; Abdoshah et al. 2022), and others reported a protective response following ocular DNA vaccination, albeit other administration routes were more effective (Li et al. 2003). The lack of expression could be due to the type of LNP that was chosen, or due migration through the nasolacrimal duct and subsequent ingestion and degradation in the gastrointestinal tract caused by the acidic pH of the crop and stomach combined with the presence of lipase in the gizzard (Witmer 1995; He et al. 2015; Kierończyk et al. 2016; Tabata et al. 2017; Ryals et al. 2020; Watanabe et al. 2020; Albawaneh et al. 2021).

The total amount of protein produced was similar after IM, SC and IO administration, but the expression pattern differed. Notably, IO administration resulted in widespread expression over the entire body, while IM and SC injection showed localized expression near the injection site. During IO administration, the saRNA is injected directly into the amniotic fluid (Ricks et al. 1999; Wakenell et al. 2002; Avakian 2006), which is absorbed by the embryo through various routes, explaining the more diffuse expression pattern. By contrast, IM and SC injection resulted in a more localized luciferase expression pattern near the injection site. Despite this difference in expression pattern, all successful routes exhibited comparable expression kinetics, which is surprising as the earliest possible imaging moment for the hatched chicks in the IO group (eggs injected on day 18 of embryonation) was 3 days post prime injection, and 6 h in the IM and SC groups.

Despite the weak antigenic properties of luciferase, anti-luciferase antibodies were detected after IM, SC and IO administration, but not following ocular administration. The higher antibody titers following prime IO vaccination may be due to the 3-day earlier administration, compared to the IM and SC group (Apanius et al. 1997; Pereira et al. 2019). However, two weeks after the subsequent SC booster vaccination, the seroconversion rate dropped, despite the luciferase expression in the IO group being the highest of all groups at the time of booster administration. The weak booster-response could be due to establishment of mainly mucosal (memory) immune responses and a high antibody turn-over after the first IO injection (Leslie and Clem 1969; Higgins 1976; Alizadeh et al. 2017), or because the body experienced the booster shot as sustained antigen exposure instead. In the IM and SC groups, the seroconversion rate increased to almost 100% two weeks after the boost, but the mean antibody titers remained relatively unchanged. In general, weak booster effect might be explained by the use of a new luciferase protein batch for the coating of the ELISA plates used for sera of boosted chickens. Additionally, the very low immunogenicity of luciferase has been reported to hamper detection of a boost effect by others as well (Tammemagi et al. 1995; Cook 2000; Caponi et al. 2020).

Dose-titration showed strong luciferase expression with all doses (0.20, 1.0, 5.0 µg), but the dose-effect differed between SC and IM administration routes. Whereas, the 0.20 µg dose achieved a similar expression level *via* both routes, an increase in expression with both higher doses was only observed after IM administration. The absent increase in the SC group can be due to the presence of higher amounts of innate immune cells, such as dendritic cells and macrophages, in the skin than in the muscle (Tammemagi et al. 1995; Richmond and Harris 2014). These cells highly express pattern recognition receptors (PRRs), like toll-like receptors (TLRs), and retinoic acid-inducible gene-I-like receptors (RIG-I-like receptors) in some poultry species, which sense the presence of non-self RNAs and subsequently activate dsRNA-dependent protein kinase (PKR) and 2′-5′-oligoadenylate synthetase (OAS) leading to either inhibit mRNA translation or induce mRNA degradation (Zhong et al. 2018; Linares-Fernandez et al. 2020; Sid et al. 2023). Hence, a high dose of saRNA-LNPs are more likely to intensely trigger the innate immune response when administered SC than after IM injection.

Single-shot SC or IM vaccination resulted in increased antibody titers and seroconversion rates over time, suggesting that maximal antibody titers are not reached within the initial two-week period, which correlates with the sustained high luciferase expression two weeks post-prime injection. Furthermore, these data imply that a prime-boost interval of more than two weeks might be more advantageous. While single-shot vaccination appears to be effective, a booster dose resulted in enhanced antibody titers and seroconversion rates. After IM prime-boost, all tested dosages resulted in 100% seroconversion. Moreover, in line with the dose-dependent luciferase expression, the results indicate that saRNA vaccination in poultry using as little as 0.20 µg LNP-formulated saRNA leads to equipotent (IM) or higher (SC) antibody response than 5.0 µg saRNA-LNPs. Thus, the use of luciferase as a model antigen allowed us demonstrate that antibody titers do not always positively correlate with antigen expression levels.

Next, we IM vaccinated broiler chickens with 1.0 µg saRNA encoding an immunodominant antigen, the hemagglutinin head-domain of H5N1 (Anhui) avian influenza. After single-shot vaccination, the HI titers were, respectively, 44, 60 and 44 at 2, 4 and 6 weeks after vaccination. Boosting two weeks after the prime increased the mean HI titers to 320 and 264 at week 4 and 6, respectively. In the boosted group, all chickens displayed titers above 40 at every measured time point. According to the World Organization for Animal Health (WOAH), HI titers of 32 or higher indicate protection against mortality, whereas titers of 40 or higher indicate protection against viral shedding (Terrestrial Animal Health Code 2023). Some chickens in the single-shot vaccinated group had titers of 16 at 6 weeks, indicating that these could be susceptible to the virus. However, a recent study that compared vectored vaccines to inactivated vaccines found a good protective immunity at lower HI titers, which was attributed to increased cellular immunity induced by vectored vaccines (Mo et al. 2023). mRNA vaccines are also known to elicit a strong cellular immune response, yet due the limited number of trials employing mRNA vaccines, it is not yet clearly determined whether they achieve a similar level of protection with lower HI titers compared to inactivated vaccines (Mo et al. 2023; Boretti 2024). Nevertheless, a recent study utilizing an H5-encoding saRNA vaccine in ducks, which demonstrated 100% survival at HI titers of 16, appears to support this hypothesis (Ministère de l’agriculture et de la souveraineté alimentaire F 2023; Cazaban et al. 2023a, 2023b).

Our results are conflicting with the recent findings of Comes et al. (Comes et al. 2024) who also investigated antibody responses in chickens after vaccination with a VEEV-based saRNA vaccine encoding the H5 antigen. In contrast to our findings, they found very low H5 antibody titers. Similar to our study, the saRNA vaccine was delivered with an ionizable LNP. However, we identified two shortcomings in the work of Comes et al. that likely explain the low efficacy of their saRNA-LNP vaccine. First, their saRNA vaccine purification strategy is suboptimal because it does not remove double-stranded (ds) RNA, a potent trigger of innate immune responses (Zhong et al. 2021). Second, Comes et al. used a 10 µg dose, whereas we use only 1 µg. High doses of saRNA are known to trigger a stronger innate immune response. In sum, based on our longstanding experience in the field of saRNA vaccines, we believe that the high dose of saRNA and the presence of dsRNA in the saRNA vaccine of Comes et al. triggered an overly strong innate immune response, which curtailed the replication and translation of the saRNA vaccine and, consequently, its efficacy.

In conclusion, the saRNA-LNP platform demonstrated both efficient *in vivo* translation after IM, SC, and *in ovo* administration, and antibody production against both luciferase and H5 in poultry. Importantly, the lowest dose of 0.2 µg induced equipotent responses and yielded the highest mean antibody titers after subcutaneous administration with no lower dose limit identified so far.

Single-shot vaccination is feasible and induces long-lasting positive HI titers, but a booster dose enhances the immune response, which could benefit further from an extended prime-boost interval. Thus, low-dose self-amplifying RNA vaccines appear to be an attractive platform for cost-effective mass vaccination in poultry.
